# Combined effects of blood pressure and glucose status on the risk of chronic kidney disease

**DOI:** 10.1038/s41440-024-01683-x

**Published:** 2024-04-26

**Authors:** Maya Toyama, Michihiro Satoh, Shingo Nakayama, Hideaki Hashimoto, Tomoko Muroya, Takahisa Murakami, Takuo Hirose, Taku Obara, Naoki Nakaya, Takefumi Mori, Takayoshi Ohkubo, Yutaka Imai, Atsushi Hozawa, Hirohito Metoki

**Affiliations:** 1 Department of Preventive Medicine and Epidemiology, Tohoku Medical Megabank Organization, Tohoku University, Sendai, Japan; 2 Division of Public Health, Hygiene and Epidemiology, Faculty of Medicine, Tohoku Medical and Pharmaceutical University, Sendai, Japan; 3 Department of Nephrology, Self-Defense Forces Sendai Hospital, Sendai, Japan; 4 Department of Pharmacy, Tohoku Medical and Pharmaceutical University Hospital, Sendai, Japan; 5 Division of Nephrology and Endocrinology, Faculty of Medicine, Tohoku Medical and Pharmaceutical University, Sendai, Japan; 6 Division of Internal Medicine, Izumi Hospital, Sendai, Japan; 7 Division of Aging and Geriatric Dentistry, Department of Rehabilitation Dentistry, Tohoku University Graduate School of Dentistry, Sendai, Japan; 8 Department of Endocrinology and Applied Medical Science, Tohoku University Graduate School of Medicine, Sendai, Japan; 9 Department of Pharmaceutical Sciences, Tohoku University Hospital, Sendai, Japan; 10 Department of Hygiene and Public Health, Teikyo University School of Medicine, Tokyo, Japan; 11 Tohoku Institute for Management of Blood Pressure, Sendai, Japan

**Keywords:** Chronic kidney disease, Blood pressure, Hypertension, Diabetes mellitus, Cohort study

## Abstract

This study aimed to assess the combined effects of blood pressure (BP) and glucose status on chronic kidney disease (CKD) incidence in young and middle-aged adults. We examined data from 1,297,341 Japanese individuals aged <60 years (60.1% men; mean age 41.4 ± 9.3 years) with no history of CKD at baseline. The interval-censored Cox proportional hazards model with covariates was used. During a median follow-up period of 2.1 years, new onset CKD (estimated glomerular filtration rate <60 ml/min/1.73 m^2^ and/or proteinuria) occurred in 80,187 participants. In participants without antihypertensive treatment (AHT), the adjusted hazard ratios (95% confidence interval) per 1-standard deviation, that is, 15 mmHg increase in systolic BP for CKD incidence, were 1.08 (1.07–1.09), 1.12 (1.10–1.13), and 1.15 (1.12–1.18) in normoglycemia, borderline glycemia, and diabetes groups, respectively. These ratios were significantly higher in the borderline glycemia and diabetes groups compared with those in the normoglycemia group (interaction *p* < 0.0001). The interaction between BP and borderline glycemia was evident when the outcome definition was restricted to proteinuria. In participants under AHT, systolic BP was most strongly associated with CKD risk in the diabetes group, although no significant interaction was observed. High BP and high glucose status may synergistically increase the incidence of CKD. Strict BP management may play an important role in the early prevention of CKD in individuals with worse glucose status within the young and middle-aged population.

**Figure:**
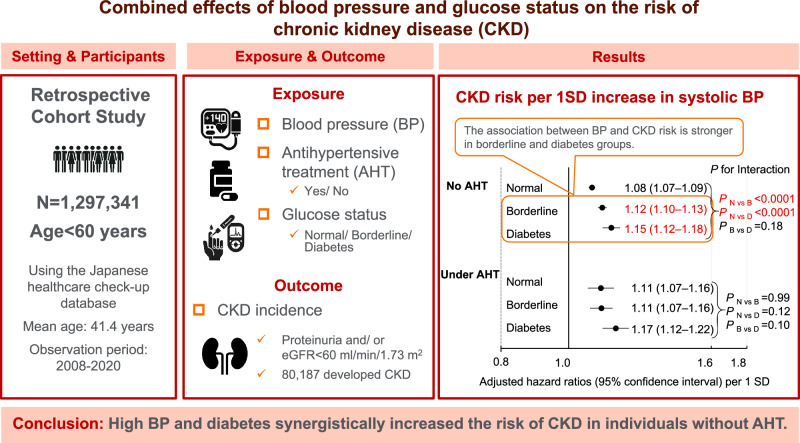
This large-scale longitudinal cohort study showed high BP and diabetes synergistically increased the risk of CKD in individuals without AHT. Strict BP management may play an important role in the early prevention of CKD in individuals with worse glucose status within the young and middle-aged population.

This large-scale longitudinal cohort study showed high BP and diabetes synergistically increased the risk of CKD in individuals without AHT. Strict BP management may play an important role in the early prevention of CKD in individuals with worse glucose status within the young and middle-aged population.

## Abstract (web summary)

This large-scale longitudinal cohort study showed high BP and diabetes synergistically increased the risk of CKD in individuals without AHT. Strict BP management may play an important role in the early prevention of CKD in individuals with worse glucose status within the young and middle-aged population.

## Introduction

Chronic kidney disease (CKD) has emerged as a significant global health burden in the past two decades. In 2017, the global prevalence of CKD was 9.1% (about 697.5 million cases), representing a 29.3% increase compared to 1990 [1]. CKD has also been recognized as an independent risk factor for cardiovascular disease, as well as a leading cause of end-stage renal disease [2]. Moreover, CKD is irreversible and is associated with a greater relative risk of death in younger than in older adults [3]. The prevalence of CKD in adults aged ≥30 years in the United States is predicted to increase [4], suggesting that the early prevention of CKD, particularly in the younger population, will become more important in the future.

Globally, hypertension and diabetes are considered the two leading drivers of CKD [5], and various studies suggested that both of them were independent risk factors in the incidence of CKD [6–8]. Hypertension and diabetes often coexist and share similar etiological and pathological mechanisms [9–11]. Thus, exploring the association between the coexistence of hypertension and diabetes and the heightened risk of CKD in the younger population may provide insights for the early prevention of future CKD incidence through effective management and treatment of these two diseases.

Studies examining the combined effects of blood pressure (BP) and glucose status on CKD incidence have yielded inconsistent results [12–15]. A previous study indicated a synergistic effect of BP and glucose status on the incidence of coronary artery disease or cerebrovascular disease [15]. Similarly, another longitudinal study involving 5823 Asians demonstrated a synergistic interaction between hypertension and diabetes in relation to CKD incidence [14]. However, the previous study categorized participants into only four groups based on the presence or absence of hypertension and diabetes, with a limited number of participants (*n* = 309) having both conditions. This limited sample size hindered a detailed assessment of the association between BP and diabetes [14]. Meanwhile, some studies have reported no significant synergistic interaction between hypertension and diabetes concerning kidney function decline [12, 13]. One possible explanation for these inconsistent findings is that these studies did not consider the use of antihypertensive treatment (AHT) among participants [12–15]. The association between BP and the risk of CKD incidence is reported to change with AHT [16].

Given these issues, our study aimed to evaluate the combined effects of BP and glucose status on CKD incidence in the young and middle-aged population, using data from a large-scale health examination. We further stratified the participants based on their use of AHT to gain insights into the association between BP and diabetes.

Point of view

- Clinical relevance
  High BP and worse glucose status have a positive synergistic interaction effect on the risk of chronic kidney disease (CKD) in individuals without antihypertensive treatment (AHT). Strict BP management may play an important role in the early prevention of CKD in individuals with worse glucose status within the young and middle-aged population.
- Future direction
  To further investigate the combined effects of BP and glucose status on CKD incidence, a nationwide survey taking into account the duration of hypertension and diabetes and information regarding the type of AHT should be conducted.
- Consideration for the Asian population
  Given the large number of people with CKD in Asian populations, urgent action may be needed in Asia for the early prevention of CKD through strict management of BP and blood glucose levels.

## Methods

### Study design and populations

The current study analyzed data from the Japan Medical Data Center (JMDC) health insurance claims database. The JMDC database contains the annual health check-up data of Japanese employees and their dependents aged <75 years, who are enrolled in large companies’ health insurance plans [16–18]. Individuals aged ≥75 years are not included in the JMDC database, as they are covered by the public health insurance system (the medical care system for the advanced elderly).

**Fig. 1:**
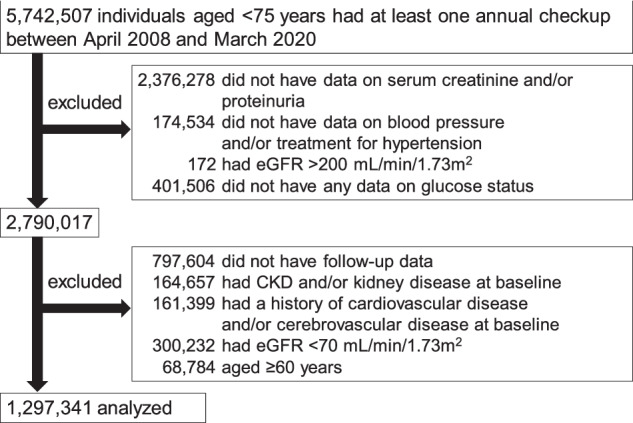
Flowchart of the participant selection process. CKD chronic kidney disease, eGFR estimated glomerular filtration rate

A flowchart illustrating the participant selection process is presented in Fig. 1. The JMDC database initially consisted of 5,742,507 individuals aged <75 years who had undergone at least one annual health check-up between April 2008 and March 2020. Among them, 2,376,278 individuals lacked serum creatinine measurements, as they are not mandatory in Japanese annual health check-ups. Additionally, 174,534 individuals without information on BP or AHT, 172 individuals with eGFR >200 ml/min/1.73 m^2^ (considered outliers), and 401,506 individuals without any information on glucose status were excluded. After the exclusion, follow-up data were available for 1,992,413 individuals. Of those, 164,657 with a history of CKD or kidney disease and 161,399 with a history of cardiovascular disease and/or cerebrovascular disease at baseline, considered to already have advanced vascular disease, were excluded [2, 19]. Moreover, we excluded 300,232 individuals with an eGFR between 60 and 69 ml/min/1.73 m^2^ due to their higher risk of CKD compared to those with eGFR ≥70 ml/min/1.73 m^2^ [16, 18, 20]. Furthermore, 68,784 individuals aged ≥60 years at baseline were excluded to focus on evaluating the CKD risk specifically in the young and middle-aged population. Finally, 1,297,341 participants were included in the present analysis.

JMDC, Inc. has received permission from the health insurance societies for the data usage. We have contracted with JMDC to use the data and have received it as anonymously processed information. According to the Ethical Guidelines for Medical and Biological Research Involving Human Subjects in Japan, obtaining approval from the Institutional Review Board or Ethics Committee and informed consent from each participant is not required in this process [21]. This exemption was due to the use of uncoupled and anonymized data from the JMDC [21].

### Data collection

We collected the data for this study at the annual health check-ups. Health check-ups in Japan are encouraged to comply with the guidelines recommended by the Japanese Ministry of Health, Labour, and Welfare [22]. The guideline recommends conducting blood tests after fasting for at least 10 h and performing urine tests by collecting fresh, midstream urine samples [22]. Methods for laboratory testing are also specified in the guideline, which recommends only the enzymatic method for measuring creatinine levels; for other items, various methods are recommended [22]. Information including smoking status, alcohol consumption, the use of antihypertensive, glucose lowering, and anti-dyslipidemia drugs, and history of cerebrovascular disease and ischemic heart disease were collected via a self-administered questionnaire. Dyslipidemia was defined as low-density lipoprotein cholesterol (LDL-C) ≥ 3.62 mmol/l (≥140 mg/dl), high-density lipoprotein cholesterol (HDL-C) < 1.03 mmol/l (40 mg/dl), triglycerides ≥1.69 (150 mg/dl), or the use of anti-dyslipidemia medications.

### Definitions

The Japanese guidelines recommend that BP be measured twice consecutively in the sitting position for annual health check-ups [23]. If two measurements were available, the average of the two measurements was used in the present study. First, we divided the participants into two groups: those not taking AHT and those taking AHT. Participants not taking AHT were further classified into five or four categories based on their systolic BP (≤119, 120–129, 130–139, 140–159, ≥160 mmHg) or diastolic BP (≤79, 80–89, 90–99, ≥100 mmHg) according to the Japanese Hypertension Society (JSH) 2019 guidelines [23]. Participants under AHT were also classified into three categories according to systolic and diastolic BP (≤129, 130–139, ≥140 mmHg and ≤79, 80–89, ≥90 mmHg), taking into account target levels of BP control [23] and the number of participants in each group. Additionally, participants were further classified as having diabetes, borderline glycemia, or normoglycemia based on fasting glucose levels, HbA1c levels, and the use of glucose lowering medications.

Diabetes was defined as having a fasting glucose level ≥126 mg/dl (7.00 mmol/l), HbA1c ≥ 6.5% (48 mmol/mol), or receiving a glucose lowering medication. Borderline glycemia was defined as fasting glucose levels between 100 mg/dl (5.6 mmol/l) and 125 mg/dl (6.9 mmol/l) and/or HbA1c levels between 5.7% (39 mmol/mol) and 6.4% (47 mmol/mol), without using a glucose lowering medication. Normoglycemia was defined as fasting glucose levels <100 mg/dl (5.6 mmol/l) and HbA1c < 5.7% (39 mmol/mol), without using a glucose lowering medication [15, 24]. HbA1c was reported in National Glycohemoglobin Standardization Program (NGSP) units (%) [25].

### Outcome and follow-up

For this study, CKD was defined as an estimated glomerular filtration rate (eGFR) < 60 ml/min/1.73 m^2^ and/or the presence of proteinuria, based on previous epidemiological studies [8, 14, 18, 20]. The eGFR was calculated using a modified version of the equation used most commonly in Japan: Japanese Society of Nephrology (JSN) eGFR (ml/min per 1.73 m^2^) = 194 × (serum creatinine)^−1.094^ × Age^−0.287^ (×0.739, if female) (hereinafter referred to as eGFR) [26, 27]. Additionally, we calculated the eGFR using the Chronic Kidney Disease Epidemiology Collaboration (CKD-EPI) equation modified for the Japanese population, incorporating the Japanese coefficient, as follows: CKD-EPI eGFR (ml/min/1.73 m^2^) = 141 × min (Cr/κ, 1)^α^ × max (Cr/κ, 1)^−1.209^ × 0.993^Age^ × 1.018 (if female) × 0.813 (Japanese coefficient). Where κ and α are 0.7 and −0.329 in females and 0.9 and −0.411 in males, respectively. In this equation, “min” indicates the minimum of SCr/κ or 1, and “max” indicates the maximum of SCr/κ or 1 [28, 29]. The presence of proteinuria was confirmed with a dipstick test for spot-urine. The results were defined as positive when the dipstick test showed 1+ or more, given that most of the individuals with dipstick 1+ or more have been found to have albuminuria [30, 31].

The baseline for this study was considered as the data recorded at the first annual health check-up. The primary outcome was the new onset of CKD confirmed during the subsequent annual check-ups. The time to CKD progression was interval-censored between the last measurement without confirmed CKD and the first measurement with confirmed CKD [32–34]. If multiple CKD events occurred during the follow-up period, only the first event was considered for analysis. In cases where no CKD event occurred during the follow-up (i.e., right censoring), the final follow-up date was the date of the final annual health check-up included in the study.

### Statistical analysis

We calculated age-standardized CKD rates using the direct method, assuming a population with the same proportion of individuals aged <40, 40–49, and ≥50 years. An interval-censored Cox proportional hazards model was used to assess the adjusted hazard ratios for CKD incidence, considering cubic splines of the baseline hazard [33].

When analyzing the adjusted hazard ratios for CKD incidence among groups according to the cross-classification based on systolic or diastolic BP and glucose status, the group with the lowest BP and normoglycemia was set as the reference. Adjusted hazard ratios were then calculated per 1 SD, that is, 15 mmHg increase in systolic BP or 11.5 mmHg increase in diastolic BP, in each glucose status for comparability between systolic and diastolic BP. Interactions between BP and borderline glycemia or diabetes vs. normoglycemia were tested using the BP× borderline glycemia category (for calculation of interaction *P*_N vs. B_) and the BP× diabetes category (for calculation of interaction *P*_N vs. D_) in the models. For interactions between BP and borderline glycemia vs. diabetes, the model included the BP × diabetes category (for calculation of interaction *P*_B vs. D_) and the BP× normoglycemia category. The schema showing the calculation of the interaction *P* is summarized in Supplementary Fig. 1.

Covariates included in the model were sex, age, body mass index (BMI), current smoking, current drinking, dyslipidemia, and eGFR at baseline. Baseline eGFR was treated as a continuous variable. We performed the same analysis with outcomes of eGFR <60 ml/min/1.73 m^2^ and proteinuria. We also restricted our analysis to participants whose baseline check-ups were conducted between 2015 and 2020 to create generalizable results for a recent Japanese population. Stratification analyses were additionally performed according to sex, age (<50 years of age and ≥50 years of age) and baseline systolic BP (<140 mmHg and ≥140 mmHg), which are strong risk factors for CKD [5, 6, 35].

Continuous variables were expressed as means ± standard deviation (SD). All analyses were conducted using SAS software (version 9.4; SAS Institute, Cary, North Carolina, USA). A *P* value < 0.05 was considered statistically significant.

## Results

### Baseline characteristics

Among the 1,297,341 participants, 779,988 (60.1%) were men. The mean values for age, BMI, systolic and diastolic BP, fasting plasma glucose (FPG), and HbA1c were 41.4 ± 9.3 years, 22.7 ± 3.7 kg/m^2^, 117.3 ± 15.3/ 72.5 ± 11.6 mmHg, 93.2 ± 16.0 mg/dl, and 5.4 ± 0.5%, respectively. The median baseline health check-up year in this study was 2018 (interquartile range 2015–2018), with most data collected between 2015 and 2018.

Table 1 shows the baseline characteristics and the number of CKD events according to a combination of five systolic BP categories and three glucose categories among participants without AHT. Within the same systolic BP category, the mean age, BMI, systolic BP, and proportions of men and smokers consistently increased with higher glucose status. The number of CKD events and the sex-and-age-adjusted incidence rate for CKD per 1000 person-years were highest in the group with the highest BP and diabetes. Similar associations were observed among participants under AHT (Supplementary Table 1).

**Table 1:** Baseline characteristics and the number of CKD events in each systolic BP-glucose status among participants without AHT

	**SBP** **≤** **119** **mmHg**			**SBP 120–129** **mmHg**			**SBP 130–139** **mmHg**		
	**Glucose status**			**Glucose status**			**Glucose status**		
**Characteristics**	**Normal**	**Borderline**	**Diabetes**	**Normal**	**Borderline**	**Diabetes**	**Normal**	**Borderline**	**Diabetes**
*N*	568,368	151,258	11,558	193,833	80,876	10,069	83,407	46,910	7724
Men, %	46.6	57.9	76.8	71.1	74.6	82.1	74.8	77.1	83.6
Age, years	38.5 ± 8.8	44.0 ± 8.1	47.2 ± 7.8	39.7 ± 9.2	44.6 ± 8.1	47.3 ± 7.6	41.4 ± 9.2	45.4 ± 8.2	47.4 ± 7.6
Body mass index, kg/m^2^	21.3 ± 2.8	22.5 ± 3.3	24.6 ± 4.1	22.9 ± 3.3	24.2 ± 3.7	26.3 ± 4.4	23.7 ± 3.6	25.0 ± 4.1	27.2 ± 4.8
Current smoker, %	22.2	28.0	41.3	28.7	32.0	40.8	30.3	32.5	39.5
Alcohol consumption, %	16.0	20.5	18.4	24.3	28.1	23.0	28.8	31.6	26.1
Systolic BP, mmHg	106.4 ± 8.4	108.1 ± 8.0	109.9 ± 7.3	124.1 ± 2.9	124.3 ± 2.9	124.5 ± 2.9	133.9 ± 2.8	134.1 ± 2.9	134.3 ± 2.9
Diastolic BP, mmHg	65.6 ± 7.9	67.7 ± 8.1	70.0 ± 7.8	75.8 ± 7.4	77.5 ± 7.1	78.5 ± 6.9	82.0 ± 7.9	83.4 ± 7.5	83.9 ± 7.3
FPG, mg/dl	87.0 ± 6.5	98.1 ± 9.1	143.7 ± 46.9	88.5 ± 6.4	100.6 ± 8.8	144.4 ± 43.4	89.2 ± 6.2	101.8 ± 8.8	146.9 ± 44.6
HbA1c, %	5.3 ± 0.2	5.7 ± 0.3	7.3 ± 1.6	5.3 ± 0.2	5.7 ± 0.3	7.3 ± 1.5	5.3 ± 0.2	5.7 ± 0.3	7.3 ± 1.5
Serum creatinine, mg/dl	0.70 ± 0.13	0.72 ± 0.13	0.72 ± 0.12	0.75 ± 0.13	0.75 ± 0.12	0.73 ± 0.12	0.76 ± 0.12	0.76 ± 0.12	0.73 ± 0.12
eGFR, ml/min/1.73 m^2^	88.4 ± 13.2	85.1 ± 11.6	88.3 ± 14.3	87.2 ± 12.5	84.8 ± 11.5	88.5 ± 14.1	86.5 ± 12.1	84.7 ± 11.4	88.4 ± 14.3
Event, *n* (Event rate 1000 person-year^a^)	28,821 (16.84)	8582 (18.26)	1038 (29.19)	10,861 (18.35)	5322 (20.97)	1158 (39.09)	5406 (20.32)	3682 (24.57)	1004 (44.05)

	**SBP 140–159** **mmHg**			**SBP** **≥** **160** **mmHg**		
	**Glucose status**			**Glucose status**		
**Characteristics**	**Normal**	**Borderline**	**Diabetes**	**Normal**	**Borderline**	**Diabetes**
*N*	33,759	26,237	5427	4686	4649	1304
Men, %	73.4	76.6	82.3	63.3	68.4	75.5
Age, years	44.0 ± 8.7	46.6 ± 7.9	47.8 ± 7.4	46.2 ± 7.8	47.9 ± 7.1	48.0 ± 7.1
Body mass index, kg/m^2^	24.4 ± 4.0	25.7 ± 4.5	27.8 ± 5.1	24.9 ± 4.6	26.4 ± 5.0	28.6 ± 5.7
Current smoker, %	31.5	32.1	37.4	31.7	30.5	35.6
Alcohol consumption, %	35.0	35.5	28.4	35.7	36.6	30.4
Systolic BP, mmHg	146.4 ± 5.3	146.8 ± 5.4	147.2 ± 5.5	168.8 ± 9.5	169.5 ± 10.1	171.3 ± 12.3
Diastolic BP, mmHg	90.9 ± 8.8	91.6 ± 8.5	91.3 ± 8.3	102.9 ± 10.7	102.9 ± 10.9	103.5 ± 11.2
FPG, mg/dl	90.0 ± 6.1	103.0 ± 8.8	148.7 ± 45.4	90.4 ± 6.0	104.5 ± 8.9	157.5 ± 50.7
HbA1c, %	5.3 ± 0.2	5.7 ± 0.3	7.3 ± 1.5	5.3 ± 0.2	5.7 ± 0.3	7.5 ± 1.7
Serum creatinine, mg/dl	0.75 ± 0.12	0.75 ± 0.12	0.73 ± 0.12	0.72 ± 0.13	0.73 ± 0.13	0.71 ± 0.13
eGFR, ml/min/1.73 m^2^	85.6 ± 11.9	84.7 ± 11.6	88.9 ± 15.1	85.0 ± 11.5	84.7 ± 11.9	89.7 ± 16.1
Event, *n* (Event rate 1000 person-year^a^)	2660 (25.42)	2374 (30.58)	793 (53.31)	437 (33.00)	503 (44.19)	217 (64.08)

Data are shown as mean ± SD for continuous variables

*AHT* antihypertensive treatment, *BP* blood pressure, *FPG* fasting plasma glucose, *eGFR* estimated glomerular filtration rate

^a^CKD incidence rates were standardized by direct method for age (<40, 40–49, and ≥50)

### CKD risks according to BP and glucose status

During a median follow-up of 2.1 years (interquartile range 1.2–4.0 years), 80,187 participants developed CKD with 25,357 experiencing eGFR <60 ml/min/1.73 m^2^, 57,887 experiencing proteinuria, and 3057 participants experiencing both eGFR <60 ml/min/1.73 m^2^ and proteinuria.

Table 2 presents the adjusted hazard ratios according to BP and glucose status. In participants without AHT, the group with systolic BP ≥ 160 mmHg and diabetes had the highest hazard ratio for CKD incidence, with a value of 3.08 compared to the group with systolic BP < 120 mmHg and normoglycemia. The risks for CKD incidence increased stepwise with higher systolic BP categories within all glucose status groups and with higher glucose status within all systolic BP category groups. In the participants under AHT, the risks for CKD were also elevated with increased systolic BP and glucose status. Similar results were obtained when all analyses were repeated using diastolic BP instead of systolic BP.

**Table 2:** Adjusted hazard ratios (95% confidence intervals) for CKD incidence according to BP-glucose status

		Glucose status		
		Normal	Borderline	Diabetes
	Systolic BP, mmHg			
No AHT	≤119	1.00 (ref)	1.02 (1.00–1.05)	1.63 (1.53–1.73)
	120–129	1.01 (0.99–1.04)	1.10 (1.07–1.14)	1.96 (1.85–2.08)
	130–139	1.09 (1.06–1.12)	1.25 (1.20–1.29)	2.14 (2.01–2.28)
	140–159	1.35 (1.30–1.41)	1.49 (1.42–1.55)	2.48 (2.31–2.67)
	≥160	1.76 (1.60–1.93)	1.93 (1.76–2.11)	3.08 (2.69–3.53)
Under AHT	≤129	1.00 (ref)	1.11 (1.02–1.20)	1.44 (1.31–1.59)
	130–139	1.05 (0.95–1.16)	1.12 (1.02–1.23)	1.76 (1.58–1.95)
	≥140	1.27 (1.15–1.41)	1.41 (1.29–1.54)	2.08 (1.88–2.30)
	Diastolic BP, mmHg			
No AHT	≤79	1.00 (ref)	1.03 (1.01–1.06)	1.70 (1.62–1.79)
	80–89	1.08 (1.05–1.11)	1.23 (1.19–1.27)	2.20 (2.09–2.32)
	90–99	1.42 (1.36–1.48)	1.55 (1.48–1.62)	2.62 (2.42–2.83)
	≥100	1.69 (1.57–1.81)	1.94 (1.81–2.08)	2.92 (2.59–3.29)
Under AHT	≤79	1.00 (ref)	1.09 (0.99–1.21)	1.55 (1.38–1.73)
	80–89	1.07 (0.97–1.18)	1.16 (1.05–1.27)	1.66 (1.50–1.85)
	≥90	1.21 (1.09–1.35)	1.37 (1.24–1.51)	2.01 (1.79–2.25)

Hazard ratios were adjusted for age, sex, body mass index, current smoking, current drinking, dyslipidemia, and eGFR at baseline

*AHT* antihypertensive treatment, *BP* blood pressure, *CKD* chronic kidney disease

### Interaction between BP levels and glucose status on CKD risks

**Fig. 2:**
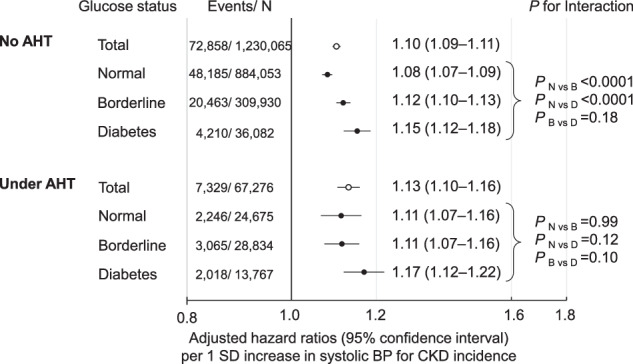
Adjusted hazard ratios (95% confidence intervals) per 1 SD increase in systolic BP for CKD incidence. Hazard ratios were adjusted for age, sex, body mass index, current smoking, current drinking, dyslipidemia, and eGFR at baseline. One SD of systolic BP is 15.0 mmHg. AHT antihypertensive treatment, BP blood pressure, CKD chronic kidney disease, SD standard deviation

Each 1 SD increase in systolic BP (15.0 mmHg) was significantly associated with an increased risk for CKD incidence in all glucose status groups, including the normoglycemia group. In participants without AHT, the adjusted hazard ratio of systolic BP for CKD incidence was significantly higher in the borderline glycemia and diabetes groups compared to those in the normoglycemia group (interaction *P*_N vs. D_ and *P*_N vs. B_ < 0.0001; Fig. 2). In participants under AHT, although the interaction was not significant, the association between systolic BP and CKD risk appeared to be clearer in the diabetes group than in the normoglycemia group (Fig. 2). Similar results were obtained when eGFR was calculated based on the CKD-EPI equation (Supplementary Fig. 2).

A similar analysis was performed using diastolic BP. In participants without AHT, the association between diastolic BP and CKD risk, similar to systolic BP, was more pronounced with worsening glucose status (interaction *P*_N vs. D_ and *P*_N vs. B_ < 0.0001; Supplementary Fig. 3). However, in participants under AHT, the interaction between diastolic BP and glucose status on CKD risk was not consistent (Supplementary Fig. 3).

### Sensitivity analyses

**Fig. 3:**
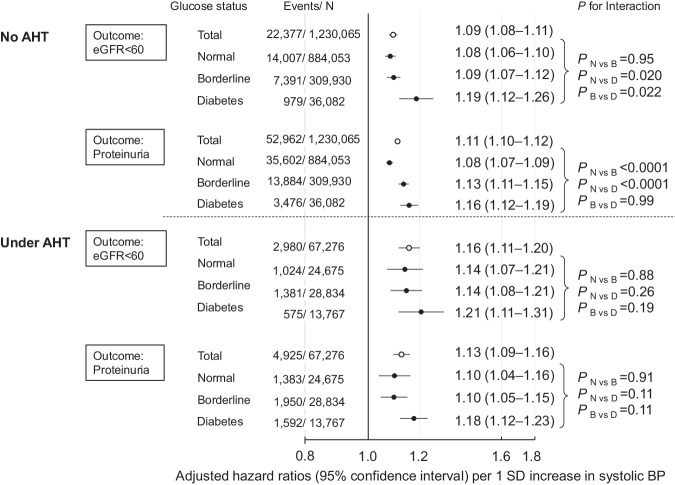
Adjusted hazard ratios (95% confidence intervals) per 1 SD increase in systolic BP for the incidence of eGFR < 60 ml/min/1.73 m^2^ and proteinuria. Hazard ratios were adjusted for age, sex, body mass index, current smoking, current drinking, dyslipidemia, and eGFR at baseline. One SD of systolic BP is 15.0 mmHg. AHT antihypertensive treatment, BP blood pressure, CKD chronic kidney disease, eGFR estimated glomerular filtration rate, SD standard deviation

When considering the outcomes of eGFR <60 ml/min/1.73 m^2^ and proteinuria separately, the results were similar to those based on the composite CKD risk except for the risk of eGFR <60 ml/min/1.73 m^2^ in the borderline glycemia group without AHT (Fig. 3). In participants without AHT, systolic BP in the borderline glycemia group was more clearly associated with proteinuria risk (interaction *P*_N vs. B_ < 0.0001; Fig. 3) rather than eGFR <60 ml/min/1.73 m^2^ risk (interaction *P*_N vs. B_ = 0.95; Fig. 3) when comparing with the normoglycemia group.

After excluding participants under AHT at baseline and during follow-up, the interaction between systolic BP and glucose status on CKD risk became more apparent (Supplementary Fig. 4). We also restricted our analysis to participants whose baseline check-ups were conducted during 2015–2020, which yielded results consistent with the main results (Supplementary Fig. 5). Participants were stratified by sex (men/women), age (<50 years/≥50 years), or baseline systolic BP (<140 mmHg/≥140 mmHg) (Supplementary Figs. 6 and 7). In participants without AHT, systolic BP was more strongly associated with CKD incidence in the borderline or diabetes groups compared to the normoglycemia group, except in the stratified group with systolic BP ≥ 140 mmHg (Supplementary Fig. 6). However, the interaction between systolic BP and glucose status tended to be inverse in the group with systolic BP ≥ 140 mmHg, although it did not reach statistical significance (Supplementary Fig. 6). In participants under AHT, the interactions between systolic BP and glucose status on CKD risk appeared to be clear in the groups of women, aged <50 years, or with systolic BP < 140 mmHg (interaction *P*_N vs. D_ ≤ 0.062; Supplementary Fig. 7).

## Discussion

Our results showed that in the general Japanese population, the CKD risk gradually increases with higher BP and glucose status, regardless of AHT. The groups with the highest BP and diabetes were at the greatest risk of CKD. Additionally, the association between BP and CKD incidence was observed even in the normoglycemia group, whereas the association was significantly stronger in the borderline glycemia or diabetes groups compared to the normoglycemia group, especially in patients without AHT.

The present study revealed that hypertension and diabetes synergistically increased the risk of CKD in individuals without AHT. It was generally considered that hypertension and diabetes were independent risk factors for CKD [6–8]. However, previous studies examining the combined effects of BP and glucose status on CKD risk have yielded conflicting results. In this regard, two prior studies involving 31,165 Chinese and 7342 Iranians did not find a significant synergistic interaction between hypertension and diabetes for reduced eGFR < 60 ml/min/1.73 m^2^ [12, 13]. Similarly, in the present study, no synergistic interactions were observed between BP and glucose status among participants under AHT. Thus, the synergistic effect of these two factors may vary depending on the therapeutic intervention and the level of hypertension. Renin-angiotensin system (RAS) inhibitors, which can prevent hypertension and CKD progression [36–40], might have obscured the synergistic effect between BP and glucose status on the CKD risk. Our results, which revealed a more synergistic interaction when the participants under AHT during follow-up were excluded, support this idea. Additionally, the disparity in the main results between Iran and our study could be due to differences in median follow-up duration (11.3 years vs. 2.1 years). Long-term therapeutic interventions for hypertension and diabetes might conceal the synergistic effects between BP and glucose status.

In our study, we evaluated the association between a 1 SD increase in BP (15.0 mmHg for systolic and 10.5 mmHg for diastolic BP) and the incidence of CKD for each glucose status (normoglycemia, borderline glycemia, and diabetes) based on data from over a million participants. This allowed for a detailed analysis of the combined effects of high BP and hyperglycemia. A recent longitudinal study in 5823 Japanese people showed a synergistic interaction of hypertension and diabetes for CKD incidence, similar to the present findings [14]. However, that study divided participants into only four groups (with or without hypertension and diabetes), limiting the assessment of CKD risk according to the degree of hypertension and the CKD risk of borderline diabetes. Additionally, the number of patients with diabetes only or diabetes accompanied by hypertension was small (258 and 309 cases, respectively), which could have been insufficient to obtain stable point estimates.

An experimental study demonstrated a synergistic interaction between hypertension and diabetes in causing kidney dysfunction and injury based on models of rats [41]. One potential mechanism for this synergistic effect is increased intraglomerular pressure, which is caused by impaired renal autoregulation and glomerular hyperfiltration [41]. Furthermore, oxidative stress and endoplasmic reticulum stress are also thought to play an important role in the synergistic effects of these two factors [41–44].

We additionally assessed the combined effects of BP and glucose status for each incidence of eGFR < 60 ml/min/1.73 m^2^ and proteinuria, respectively. The results were similar to those based on the composite CKD risk, except in participants without AHT, where the risk of eGFR <60 ml/min/1.73 m^2^ due to elevated BP did not differ between normoglycemia and borderline glycemia groups. The Framingham study reported that the risk of reduced eGFR in the borderline glycemia group could be similar to that in the normoglycemia group after adjustments for possible cardiovascular risk factors [45]. In the United Kingdom Prospective Diabetes Study (UKPDS), 30% of patients with type 2 diabetes took 15 years (median) to develop eGFR <60 ml/min/1.73 m^2^ [46]. The findings from our study, spanning a 2-year follow-up period, suggest that the synergistic effect between high BP and borderline glycemia on the risk of eGFR reduction may require a longer period to manifest. However, this may not be the case for proteinuria outcomes since the present study revealed that the association between BP and proteinuria risk was stronger in the borderline glycemia group than in the normoglycemia group.

The clear synergistic effects of systolic BP and glucose status for CKD incidence were observed in women, aged <50 years, and systolic BP < 140 mmHg, respectively. Given that men, older individuals, or those with systolic BP ≥ 140 mmHg may already have a higher CKD risk than the others [5, 6, 35], the risk of CKD could have already reached a ceiling, resulting in a weak synergistic effect of BP and glucose status on CKD risk. The strong interaction observed between systolic BP and glucose status on CKD risk, specifically among individuals with systolic BP < 140 mmHg, suggests that strict BP management may still play an important role in preventing the development of CKD in patients with diabetes, even when their BP is within levels that are not considered hypertensive or uncontrolled.

The novelty of our study lies in the detailed classification of participants according to the use of AHT and glucose status, allowing specific calculation of the risk of CKD due to elevated BP in each group. Detailed classification and accurate estimation from the large sample size of the JMDC database revealed a synergistic effect of BP and glucose status on CKD risk in participants without AHT. The association between BP and CKD risk was known to be more pronounced in individuals without AHT than in those under AHT [16], and this study suggests that prevention of diabetes may reduce the adverse effects of elevated BP on renal function.

Our study has several limitations. First, our study used only single-point creatinine and urine protein data to define CKD incidence due to the present database consisting of annual health check-ups, which do not mandate frequent measurement of serum creatinine. The current guideline defines CKD as either kidney damage or eGFR <60 ml/min/1.73 m^2^ for more than 3 months [47]. Unfortunately, we were unable to employ this criterion in our study. Furthermore, previous reports have shown that a urine dipstick test produces a considerable number of false negatives as well as a large number of false positives [48, 49]. Therefore, future studies based on quantitative measurements of urine tests are needed. Nevertheless, this method of defining CKD outcomes has been used in previous studies [8, 12–14, 16, 18, 20, 45]. Second, while the World Health Organization diagnostic criteria recommend using a 75-gram oral glucose tolerance test to determine glucose status [50], this specific laboratory data was not available in the database. Therefore, we classified the participants based on fasting glucose, HbA1c, and diabetes medication, which may have led to potential misclassification of glucose status. Third, although the Japanese guidelines specified the conditions and methods for laboratory testing and BP measurements, actual management depended on each health insurance society. Adherence to this protocol may have been limited in the present study. For the same reason, detailed information on laboratory testing methods was not available. Fourth, the data from the annual health check-ups did not include information regarding the type of AHT. Notably, a previous study based on real-world data found that AHT with angiotensin II receptor blockers had a lower risk of composite renal outcomes compared to dihydropyridine calcium channel blockers [51]. Such differences in the drug type might have influenced the results of this study among participants under AHT. Fifth, the exact duration of hypertension or diabetes in our participants was unknown. It is reported that the duration of diabetes can significantly influence the development of CKD [12, 52] and it would have been valuable to examine the results according to different duration of hypertension and diabetes. Finally, the JMDC database predominantly contains data from young and middle-aged Japanese workers employed in large enterprises. This limits the generalizability of our findings. However, previous studies using this database have yielded reasonable results regarding the association between BP and CKD risk [16, 20]. Despite these limitations, our study provides valuable evidence, particularly for the early prevention of CKD, by showcasing results in a younger population.

### Perspective of Asia

The present study, based on a large-scale Japanese health insurance claims database, suggested that high BP and worse glucose status synergistically increased the risk of CKD in individuals without AHT. The inconsistent results on the combined effects of BP and glucose status on CKD risk in Asians [12–14] may be because the combined effects of these two factors varies with the therapeutic intervention of hypertension. Up to an estimated 434.3 million adults have been reported have CKD in Asia [53]. Given the large number of people with CKD, urgent action may be needed in Asia for the early prevention of CKD through strict management of BP and blood glucose levels.

## Conclusion

Our findings showed that high BP and diabetes synergistically increased the risk of CKD in individuals without AHT. These results suggested that strict management of BP may play an important role in preventing the development of CKD in individuals with worse glucose status in the young and middle-aged population.

### Supplementary information

Supplementary information
