# Supplementary material for: Combined effects of blood pressure and glucose status on the risk of chronic kidney disease
Source: Hypertens Res. 2024 Apr 26;47(7):1831–41. doi: 10.1038/s41440-024-01683-x (PMC11224015; doi:10.1038/s41440-024-01683-x)
Supplement: Supplementary file 1 — Supplementary information [file 41440_2024_1683_MOESM1_ESM.pdf]

Supplementary Information

Supplemental to:

Combined effects of blood pressure and glucose status on the risk of chronic kidney disease

Supplementary Table 1. Baseline characteristics and the number of CKD events in each systolic BP-glucose status among participants under AHT

	SBP ≤129 mmHg			SBP 130–139 mmHg			SBP ≥140 mmHg		
	Glucose status			Glucose status			Glucose status		
Characteristics	Normal	Borderline	Diabetes	Normal	Borderline	Diabetes	Normal	Borderline	Diabetes
N	12,367	13,466	6,233	6,632	7,944	3,749	5,676	7,424	3,785
Men, %	69.5	75.6	83.7	71.6	76.8	83.5	70.1	74.8	81.4
Age, years	49.4±6.4	51.2±5.6	51.0±5.8	49.3±6.4	50.9±5.8	51.0±5.8	49.2±6.6	50.5±6.1	50.9±6.1
Body mass index, kg/m^2	24.0±3.7	25.7±4.1	27.9±4.8	24.8±4.0	26.3±4.4	28.6±4.9	24.9±4.2	26.7±4.7	28.9±5.3
Current smoker, %	28.6	30.4	37.0	26.4	28.0	33.8	28.6	28.2	31.7
Alcohol consumption, %	37.5	38.5	28.6	39.5	39.7	29.3	40.3	39.6	32.6
Systolic BP, mmHg	119.2±7.9	119.7±7.6	119.5±7.7	134.3±2.9	134.3±2.9	134.3±2.9	150.0±9.7	150.1±9.9	150.9±10.7
Diastolic BP, mmHg	77.2±8.1	77.6±8.0	76.4±7.9	85.6±7.1	85.3±7.0	84.2±7.1	93.6±9.2	93.0±9.1	91.9±9.7
FPG, mg/dL	90.2±6.1	103.1±9.1	136.9±33.2	90.6±5.8	103.7±8.8	139.7±33.9	90.6±5.9	104.5±9.0	145.7±38.7
HbA1c, %	5.3±0.2	5.7±0.3	7.0±1.1	5.3±0.2	5.7±0.3	7.1±1.1	5.3±0.2	5.7±0.3	7.2±1.2
Serum creatinine, mg/dL	0.73±0.12	0.74±0.12	0.74±0.11	0.74±0.12	0.74±0.12	0.73±0.11	0.73±0.12	0.73±0.12	0.72±0.12
eGFR, mL/min/1.73m^2	83.1±10.9	82.6±10.4	85.9±12.7	83.6±10.8	83.3±10.9	86.7±13.2	84.0±11.1	83.8±11.1	87.3±13.7
Event, n	1,003	1,260	751	605	815	579	638	990	688
(Event rate 1000 person-year*)	(30.06)	(37.90)	(47.27)	(30.54)	(31.83)	(58.35)	(41.79)	(49.00)	(79.43)

Data are shown as mean±SD for continuous variables. AHT, antihypertensive treatment; BP, blood pressure; FPG, fasting plasma glucose; eGFR, estimated glomerular filtration rate. *CKD Incidence rates were standardized by direct method for age (<40, 40–49, and ≥50).

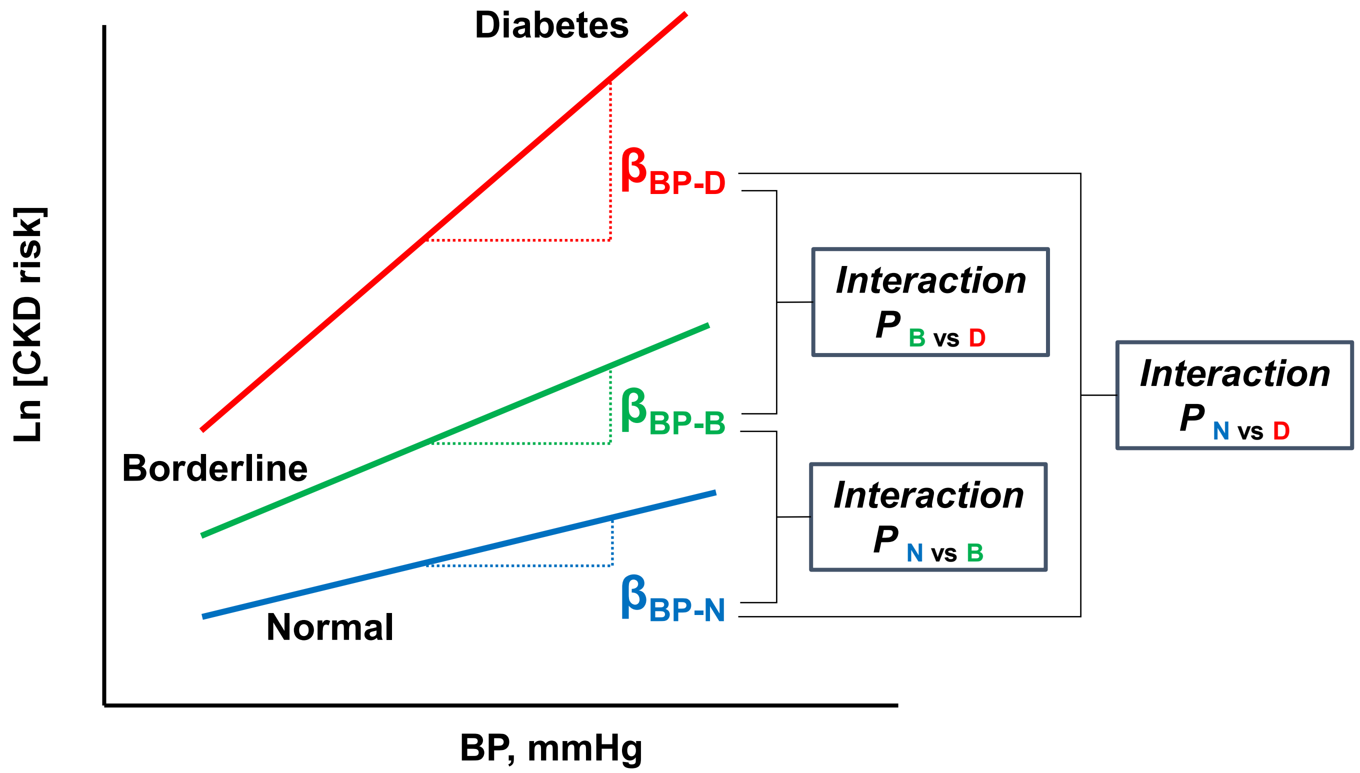

Supplementary Figure 1. Schema showing the calculation of the interaction *P*

BP, blood pressure; CKD, chronic kidney disease; SD, standard deviation

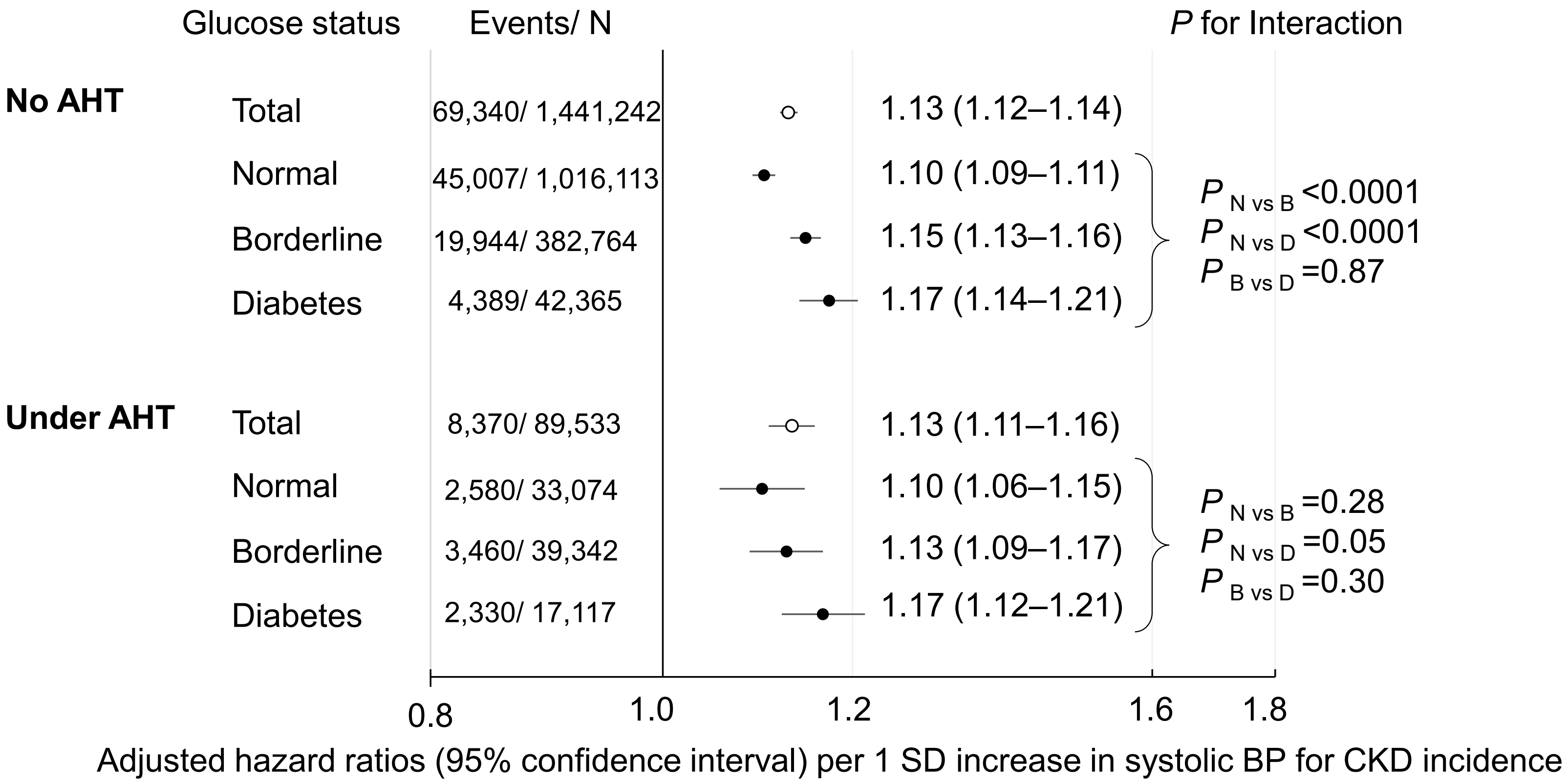

Supplementary Figure 2. Adjusted hazard ratios (95% confidence intervals) per 1 SD increase in systolic BP for CKD incidence when using CKD-EPI eGFR

Hazard ratios were adjusted for age, sex, body mass index, current smoking, current drinking, dyslipidemia, and eGFR at baseline. The eGFR was calculated using the Japanese coefficient-modified CKD-EPI equation. One SD of systolic BP is 15.0 mmHg. AHT, antihypertensive treatment; BP, blood pressure; CKD, chronic kidney disease; SD, standard deviation; CKD-EPI, chronic kidney disease epidemiology collaboration.

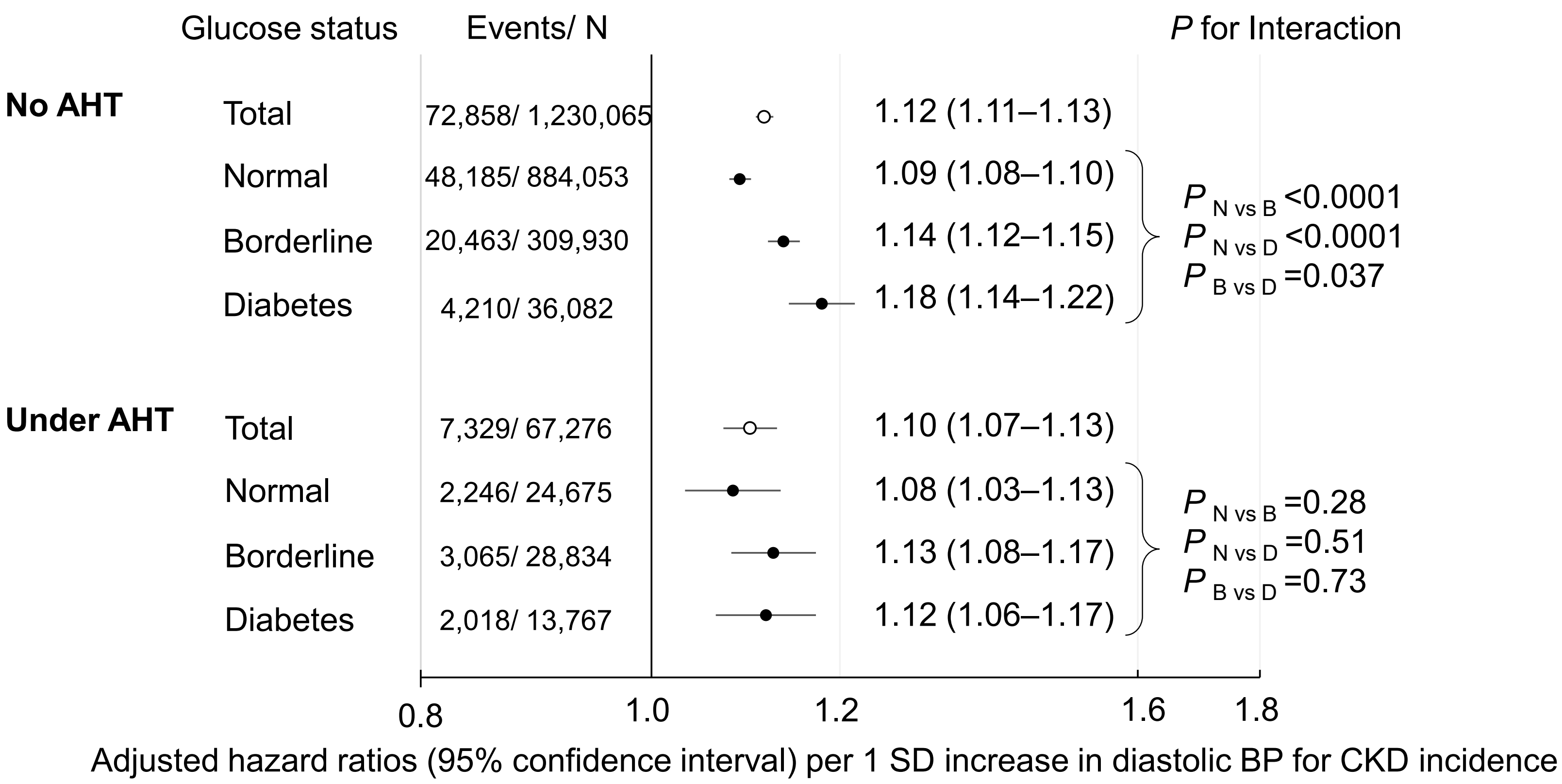

Supplementary Figure 3. Adjusted hazard ratios (95% confidence intervals) per 1 SD increase in diastolic BP for CKD incidence

Hazard ratios were adjusted for age, sex, body mass index, current smoking, current drinking, dyslipidemia, and eGFR at baseline. One SD of diastolic BP is 11.5 mmHg. AHT, antihypertensive treatment; BP, blood pressure; CKD, chronic kidney disease; SD, standard deviation.

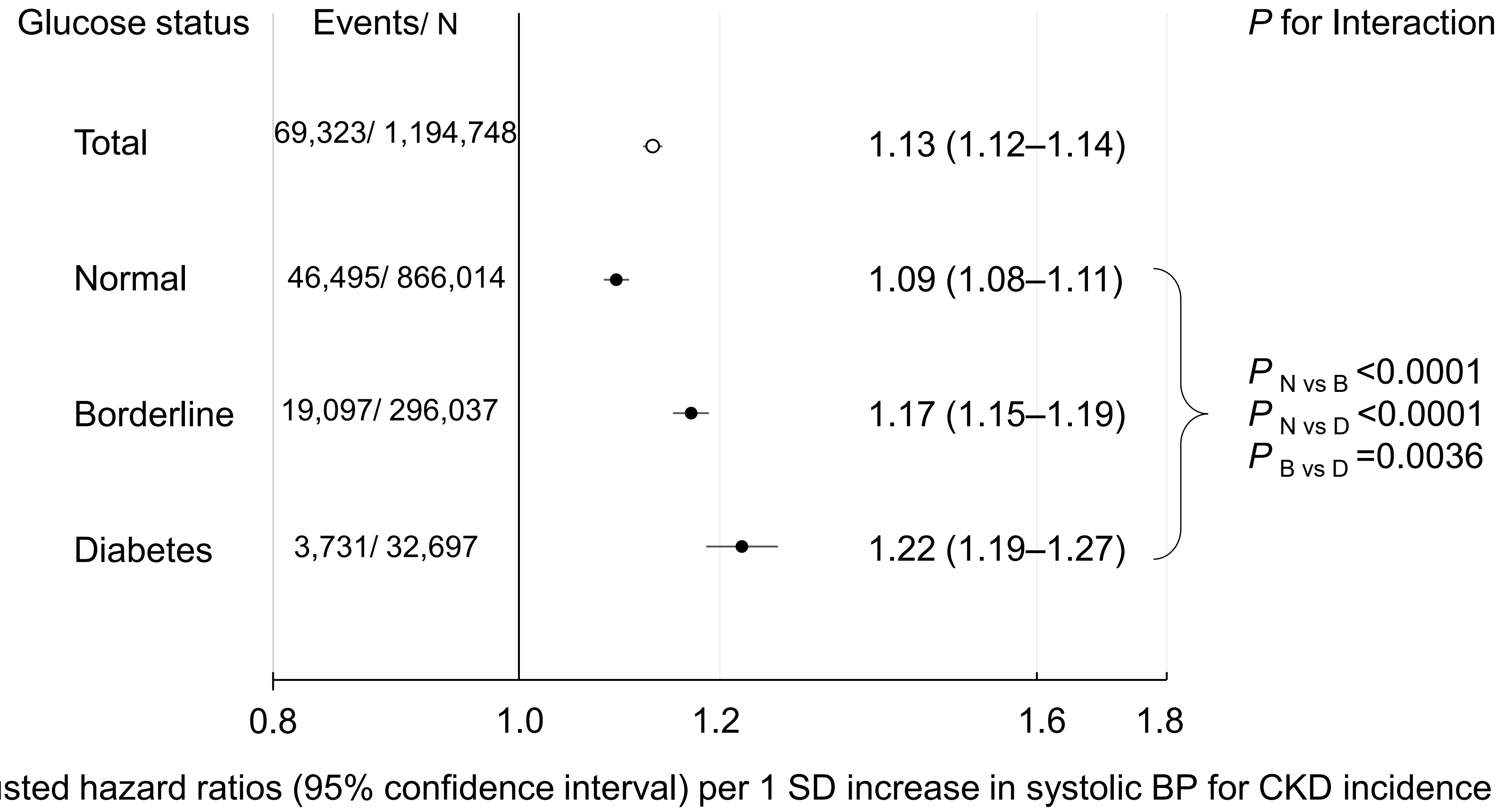

Supplementary Figure 4. Adjusted hazard ratios (95% confidence intervals) per 1 SD increase in systolic BP for CKD incidence after excluding participants under AHT at baseline as well as during follow-up

Hazard ratios were adjusted for age, sex, body mass index, current smoking, current drinking, dyslipidemia, and eGFR at baseline. One SD of diastolic BP is 11.5 mmHg. AHT, antihypertensive treatment; BP, blood pressure; CKD, chronic kidney disease; SD, standard deviation.

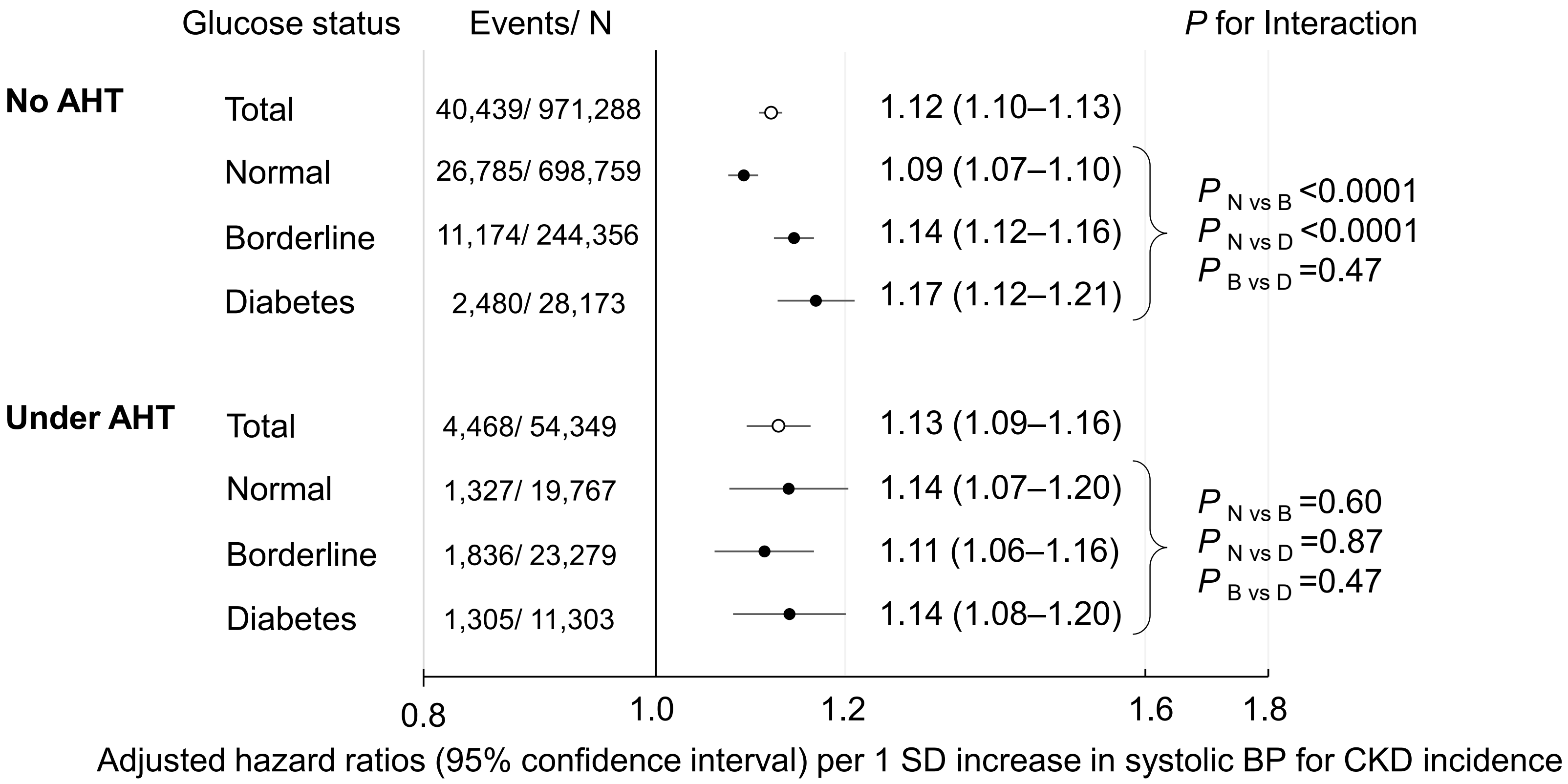

Supplementary Figure 5. Adjusted hazard ratios (95% confidence intervals) per 1 SD increase in systolic BP for CKD incidence when restricted to participants whose baseline check-ups were conducted between 2015 and 2020

Hazard ratios were adjusted for age, sex, body mass index, current smoking, current drinking, dyslipidemia, and eGFR at baseline. One SD of systolic BP is 15.0 mmHg. AHT, antihypertensive treatment; BP, blood pressure; CKD, chronic kidney disease; eGFR, estimated glomerular filtration rate; SD, standard deviation.

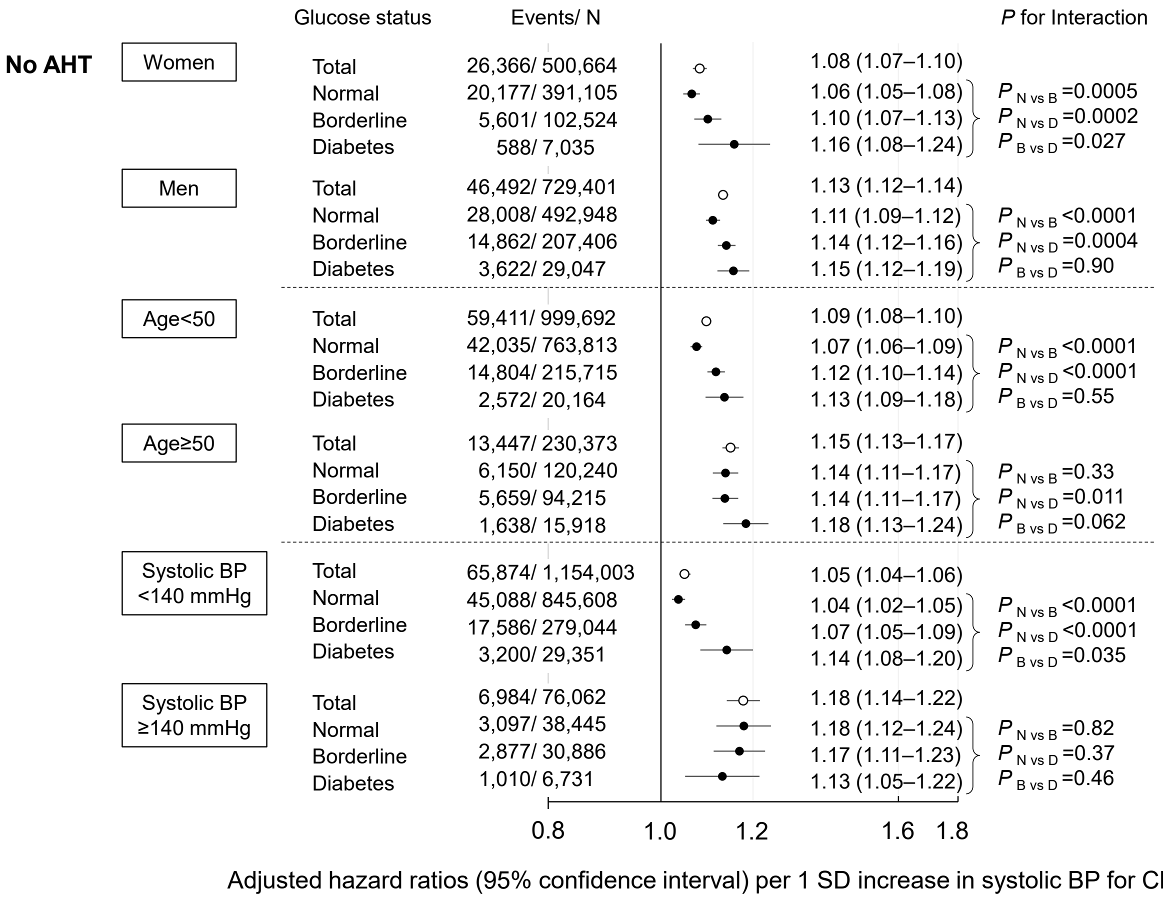

Supplementary Figure 6. Stratification analysis by sex or age or baseline systolic BP in the participants without AHT

The outcome is adjusted hazard ratios (95% confidence intervals) per 1 SD increase in systolic BP for CKD incidence.

Hazard ratios were adjusted for age, sex, body mass index, current smoking, current drinking, dyslipidemia, and eGFR at baseline.

One SD of systolic BP is 15.0 mmHg. AHT, antihypertensive treatment; BP, blood pressure; CKD, chronic kidney disease; eGFR, estimated glomerular filtration rate; SD, standard deviation.

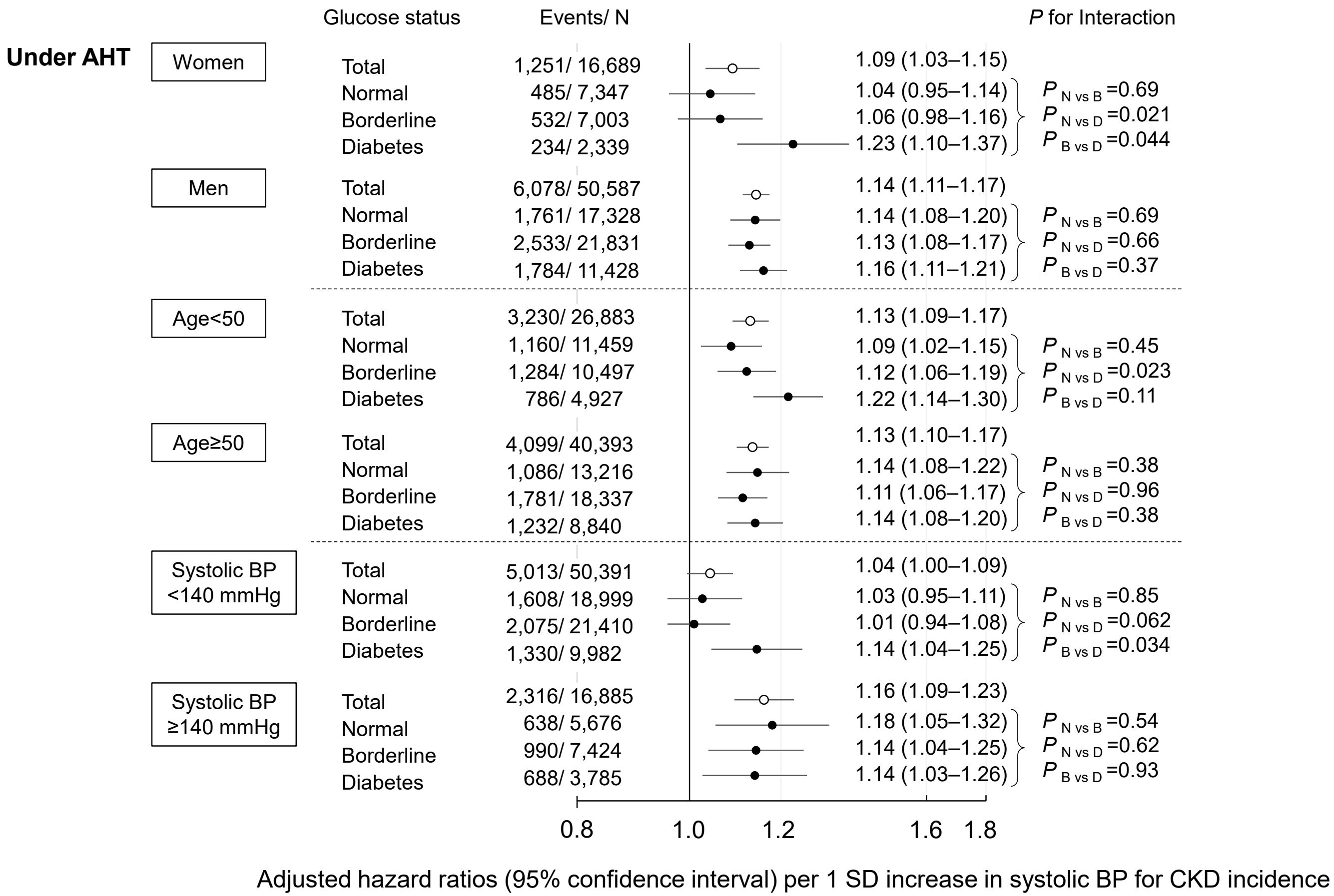

Supplementary Figure 7. Stratification analysis by sex or age or baseline systolic BP in the participants under AHT

The outcome is adjusted hazard ratios (95% confidence intervals) per 1 SD increase in systolic BP for CKD incidence.
Hazard ratios were adjusted for age, sex, body mass index, current smoking, current drinking, dyslipidemia, and eGFR at baseline.
One SD of systolic BP is 15.0 mmHg. AHT, antihypertensive treatment; BP, blood pressure; CKD, chronic kidney disease; eGFR, estimated glomerular filtration rate; SD, standard deviation.
